# Single crystalline superstructured stable single domain magnetite nanoparticles

**DOI:** 10.1038/srep45484

**Published:** 2017-03-30

**Authors:** Victoria Reichel, András Kovács, Monika Kumari, Éva Bereczk-Tompa, Emanuel Schneck, Patrick Diehle, Mihály Pósfai, Ann M. Hirt, Martial Duchamp, Rafal E. Dunin-Borkowski, Damien Faivre

**Affiliations:** 1 Department of Biomaterials, Max Planck Institute of Colloids and Interfaces, Science Park Golm, 14424 Potsdam, Germany; 2 Ernst Ruska-Centre for Microscopy and Spectroscopy with Electrons and Peter Grünberg Institute, Forschungszentrum Jülich, 52425 Jülich, Germany; 3 Institute of Geophysics, ETH-Zürich, Sonneggstrasse 5, CH-8092 Zürich, Switzerland; 4 Department of Earth and Environmental Sciences, University of Pannonia, Egyetem u. 10, H8200 Veszprém, Hungary

## Abstract

Magnetite nanoparticles exhibit magnetic properties that are size and organization dependent and, for applications that rely on their magnetic state, they usually have to be monodisperse. Forming such particles, however, has remained a challenge. Here, we synthesize 40 nm particles of magnetite in the presence of polyarginine and show that they are composed of 10 nm building blocks, yet diffract like single crystals. We use both bulk magnetic measurements and magnetic induction maps recorded from individual particles using off-axis electron holography to show that each 40 nm particle typically contains a single magnetic domain. The magnetic state is therefore determined primarily by the size of the superstructure and not by the sizes of the constituent sub-units. Our results fundamentally demonstrate the structure – property relationship in a magnetic mesoparticle.

Magnetite is a ubiquitous iron oxide mineral that is found on Earth and in planetary settings, as well as in the biological world^1^,^2^,^3^,^4^,^5^. Magnetite nanoparticles have numerous industrial and technological applications^6^,^7^,^8^,^9^, which often require well-defined magnetic behaviour. The magnetic properties of magnetite are dependent on Néel relaxation^10^, which in turn depends on particle size, morphology and interparticle interactions, as well as on the temperature and time-scale of the measurement^11^,^12^. For example, for a measurement time of 100 ms at room temperature, isolated equi-dimensional magnetite particles that have diameters of less than approximately 25 nm are superparamagnetic (SP), i.e., the directions of their magnetic moments change due to thermal fluctuations on the order of the measurement time. Particles with diameters of approximately 25 to 80 nm are typically magnetized homogeneously and considered to be stable single domain (SSD), i.e., thermally blocked, magnetic states with uniform magnetization. Particles that are larger than 80 nm are usually multi-domain (MD)^13^, i.e. each particle is split into magnetic domains.

Synthesized magnetite nanoparticles are generally considered to be individual units. However, magnetite superstructures have been reported in the literature, including 30 μm microdisks composed of 20 nm building blocks (i.e., on the SP/ SSD threshold)^14^ and 200 nm hollow spheres consisting of 40 to 50 nm aligned nanoparticles^15^, both exhibiting thermally blocked states. These types of particles are based on sub-units that are potentially too large to display SP behaviour, while the particles themselves are too large to display SSD behaviour. In a study of 174 nm particles composed of misaligned 10.9 nm nanocrystals^16^, the magnetic properties were reported to be reminiscent of SP particles, i.e., of those of the sub-units as expected in a case where the nanocrystals are not aligned in the particles. In addition, it has been shown that even a very thin non-magnetic film of about 1 nm would break the exchange interaction between two magnetic films^17^,^18^. Therefore, it remains an unsolved problem how to form monodisperse SSD magnetite from an aqueous-based process and whether these particles can form from SP building blocks. The ability to synthesize such particles will not only provide a model system of such SSD particles; it will also allow testing how such particles perform in numerous biomedical or engineering applications that require SSD particles. In this study, we applied a synthesis method that yields monodisperse SSD magnetite particles made up of SP building blocks.

It has been demonstrated that synthetic magnetite can form from aqueous solution at room temperature *via* nanoparticulate intermediates^19^. While such processes lead to the formation of SSD particles, imaging by transmission electron microscopy (TEM) has shown the particles to be widely dispersed in size due to aggregation^20^. The use of additives was reported to control the mechanism of magnetite formation and possibly the resulting nanoparticle dimensions^21^,^22^,^23^,^24^,^25^. Here, we show that the addition of polyarginine mostly leads to the formation of 40 nm monodisperse magnetite particles that are made from 10 nm sub-domains, yet possess true SSD properties.

## Results

### Structural properties

**Figure 1:**
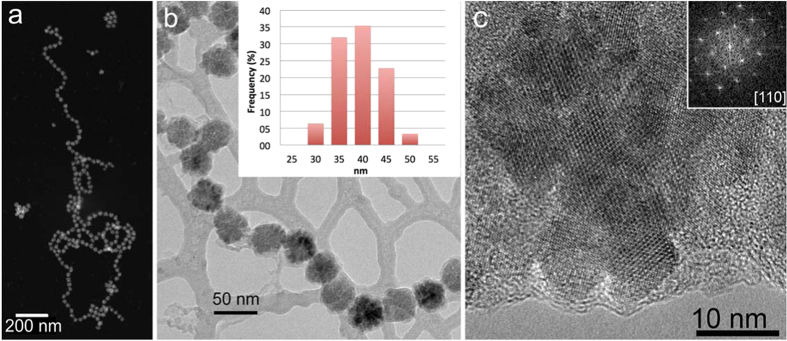
(**a**) HAADF STEM and (**b**) BF TEM images of magnetite particles assembled into chains. The particles display light and dark contrast in (**a**) and (**b**), respectively. The web-like feature in the background in (**b**) is a lacey C support film. The inset in (**b**) shows the particle size distribution, as measured from TEM images. (**c**) HRTEM image of a magnetite particle recorded with the electron beam parallel to the [110] direction of magnetite. The Fourier transform of the image is shown in the inset and indicates that, even though the particle consists of several smaller crystallites, it has the diffraction pattern of a single crystal.

TEM was used to study the sizes, shapes, structures and compositions of the particles, as well as the crystallographic orientations of individual nanocrystals within particles. The nanoparticles were found to distribute themselves into two-dimensional clusters or self-organized chains when deposited on TEM grids (Fig. 1a and b). A total of 263 particles were measured. The measured size distribution of the particles indicates that they are monodisperse, with a mean diameter of 36.7 ± 3.7 nm (inset to Fig. 1b). The size range of the particles is similar to that of magnetosomes from magnetotactic bacteria, which are often used as a reference for monodispersity^26^. BF TEM images show that an amorphous material (presumably the polyarginine) surrounds the crystals and that the particles have irregular shapes (Fig. 1b).

Elemental analysis of the nanoparticles confirmed that they are made of Fe and O, with some additional C (Supporting Information, Figs S1 and S2). The 40 nm particles have irregular, angular outlines with re-entrant angles, suggesting that they are composed of sub-units, as seen in both BF TEM and higher-magnification HAADF STEM images (Figs 1b and S1a). HRTEM images confirm that each 40 nm particle is composed of smaller sub-units, as indicated by the distinct contrast (typically bright) along their boundaries and by the presence of different HRTEM contrast in the parts that make up the entire particle and have variable thicknesses (Figs 1c, S5 and S6). Electron tomographic reconstructions confirm the irregular shapes of the particles, and slices extracted from the tomographic reconstructions reveal ~1-nm gaps within the particles, at the boundaries of their sub-units (Fig. S4). Both SAED patterns obtained from individual particles (Fig. S5) and Fourier transforms of their HRTEM images (inset to Fig. 1c and Fig. S6) are similar to single-crystal-like diffraction patterns for the entire 40 nm particles, suggesting a uniform crystallographic orientation of the subunits (despite the gaps between them). The aggregate nature of the particles is further supported by SANS, which shows a distinct feature at a *q*-value corresponding to approximately 10 nm (Fig. S7). Thus, the particles can be regarded mesocrystals^27^. High resolution X-ray diffraction revealed the lattice parameter *a* of our sample to be equal to 8.3853 +/− 0,0001 Å, which is in good agreement with the lattice parameter standard magnetite (*a* = 8.396 Å) and further away from that of maghemite (*a* = 8.347 Å)^28^.

### Magnetic properties

**Figure 2:**
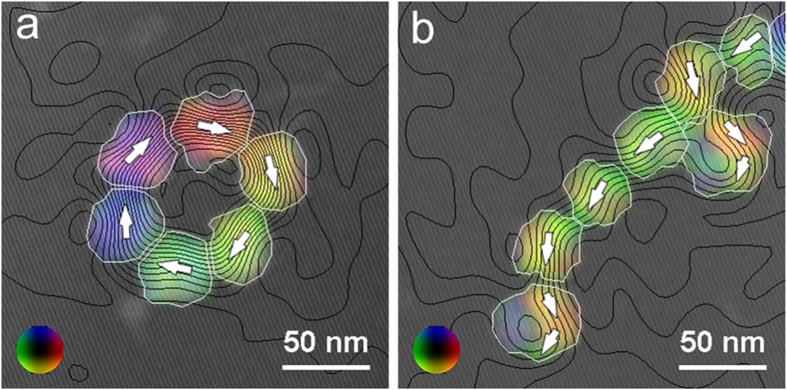
Magnetic induction maps recorded in magnetic-field-free conditions using off-axis electron holography from (**a**) a ring and (**b**) a chain of magnetite particles. The colours and contours show the direction and strength of the projected in-plane magnetic flux density, respectively, and are superimposed on a combination of the mean inner potential contribution to the phase and an off-axis electron hologram. A colour wheel is shown as an inset at the lower left corner of each image. The magnetic phase contour spacing is 2π/256 radians in each image. The white arrows indicate the direction of the magnetic induction in each particle. A thin white line marks the outer edge of each particle.

We used off-axis electron holography (EH) in the TEM to study the magnetic induction associated with individual magnetite particles arranged in rings and chains, in order to examine the magnetization states within the mesocrystals and interactions between them. A representative magnetic induction map recorded from a ring of six magnetite particles demonstrates that each particle contains a single magnetic domain (Fig. 2a), while the ring configuration of the particles constrains the magnetic field to form a flux-closed state. A similar magnetic induction map of a chain configuration also indicates that most of the particles are single magnetic domains, with the direction of the magnetic induction now determined by the overall direction of the chain (Fig. 2b). Two of the largest (ca. 50 nm) particles that are visible in Fig. 2b, however, appear to have multi-domain states, as suggested by the changes in the directions of the contour lines and colours within them. Either the subunits within these two particles (at the lower end and to the right of the chain) are not perfectly aligned, or the particles are larger than the maximum size for which such mesocrystals may still be single magnetic domains. Alternatively, we cannot exclude that the multidomain features are artefacts resulting from incomplete magnetic reversal during the electron holographic analysis.

Phase information obtained using off-axis EH can be also used to measure both the magnetic and the mean inner potential (MIP) contribution to the phase quantitatively^29^. The magnetic phase shift across each particle in the ring is approximately 0.34 radians, which is slightly smaller than the value of 0.50 radians predicted for a uniformly-magnetized 40 nm sphere of magnetite. In the case of a homogeneous material, the MIP contribution to the phase is proportional to the particle thickness in the electron beam direction. For a spherical 40 nm particle of magnetite, assuming a MIP of 17 V and an accelerating voltage of 300 kV, the MIP at the centre of the particle relative to its surroundings is predicted to be 4.4 radians. For the particles shown in Fig. 2, the measured MIP contribution to the phase varies between 2.9 and 4.0 radians, suggesting that the particles have a density that is slightly lower than that of pure magnetite. The HRTEM and STEM images show small gaps and perhaps organic material between the individual sub-units that make up each particle. As a result, both the MIP contribution and the magnetic contribution to the phase are lower than they would be for single crystals of magnetite of the same size.

**Figure 3:**
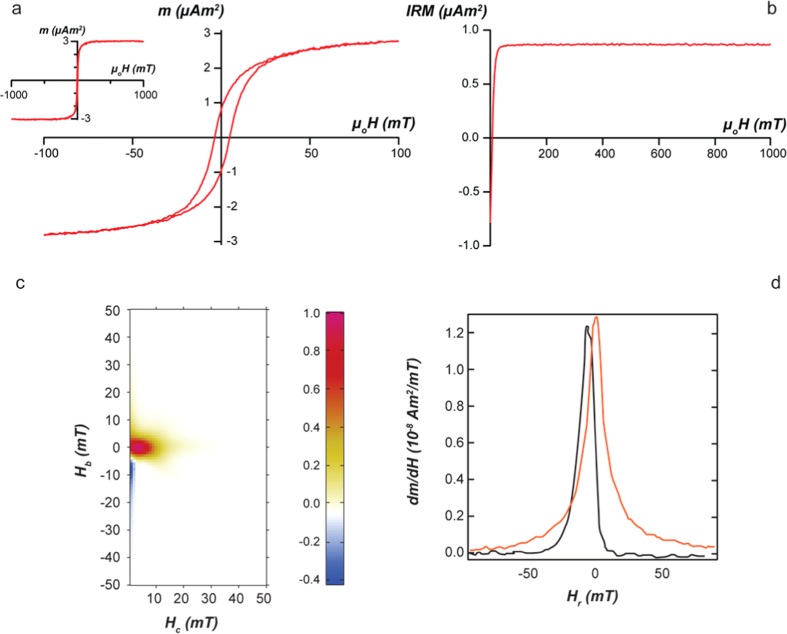
Bulk magnetic characterization of magnetite particles. (**a**) Hysteresis loops with the inset showing the saturation attained at high field; (**b**) Backfield IRM; (**c**) FORC diagram with a smoothing factor of 2; (**d**) Derivative of the magnetic moment of the reversible (blue curve) and irreversible (black curve) part of the induced magnetization.

We also analysed the bulk magnetic properties of the sample. The nanoparticles showed a thin hysteresis loop, with a coercivity *H*_*c*_ of 4.2 mT. The magnetization is saturated by 200 mT, while the ratio of saturation remanent magnetization (*M*_*rs*_) to saturation magnetization (*M*_*s*_) is 0.29 (Fig. 3a). A backfield isothermal remanent magnetization (IRM) curve is saturated by approximately 150 mT. These results indicate that the synthesized magnetite is pure and shows no significant degree of oxidation (Fig. 3b). This is confirmed by the susceptibility measurement as a function of temperature showing a Verwey transition (Fig. S11). The First Order Reversal Curve (FORC) distribution shows a spread along the coercivity axis from zero to approximately 20 mT and the presence of small closed contours in the vicinity of the origin (Fig. 3c). The fact that the distribution is narrow and very close to the origin is consistent with the presence of smaller particles, which would also be responsible for lowering the bulk coercivity and magnetization ratio from what is expected for pure SSD magnetite. A significant contribution from multi-domain particles would also lead to a broader FORC distribution at the origin. Moreover, decomposition of the FORC measurements into reversible and irreversible components of induced magnetization suggests that ca. 25% of the magnetic behaviour can be attributed to the presence of SP particles and possibly MD grains, the rest being SSD (Fig. 3d)^30^. The presence of SP particles is further supported by the measurement of magnetic hysteresis, which shows an increase in coercivity at lower temperature (Fig. S8).

The results of the FORC measurements are consistent with the TEM observations, which indicate the presence of particles that are only a few nm in size (e.g., in the boxed area in Fig. S1d) and therefore SP. In contrast, the larger magnetite particles contain single magnetic domains, as demonstrated by most of the crystals in the magnetic induction maps recorded using EH. The aligned individual sub-units within each particle interact magnetically, so that they can be treated as single magnetic entities. Each 40 nm magnetite particle can therefore be regarded as a “magnetic mesoparticle”. It is composed of a number of SP sub-units but its magnetic properties are determined by the size of the superstructure, rather than that of its components, reminiscent of the structural properties of mesocrystals^31^.

## Discussion

The magnetic properties of the particles are compatible with Fe_3_O_4_ or the slightly oxidized form γ-Fe_2_O_3_. High resolution X-ray diffraction confirms such possibility of a slightly oxidized magnetite. The Fourier transform of the high resolution TEM image in Fig. 1c, however, suggests that only pure magnetite is present. Although we cannot rule out that some of the superstructures contain maghemite, this would only lead to a slight decrease in their total magnetization, but does not alter the fact that the particles display the properties of a single structure.

**Figure 4:**
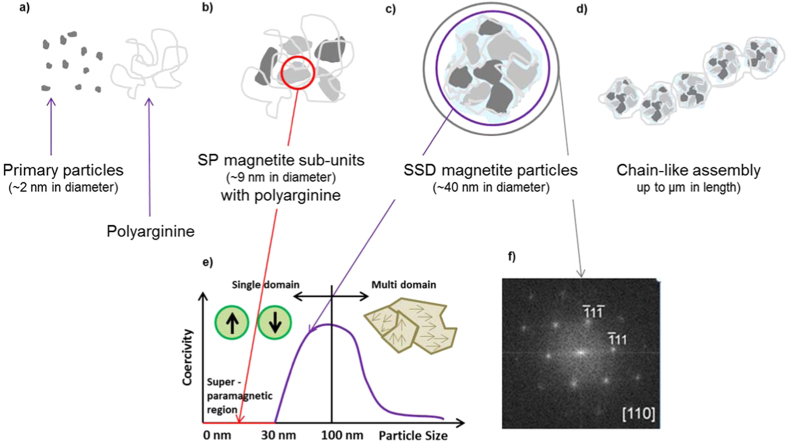
Schematic diagram of the proposed mechanism of formation of the SSD particles from SP sub-units and their ability to form chain structures. The steps involve: (**a**) nucleation of initial primary particles and their agglomeration, leading to; (**b**) formation of 9 nm magnetite sub-units; (**c**) these sub-units are assembled into larger particles by the additive and (**d**) assemble into chains, possibly resulting from the fact that (**e**) each particle exhibits SSD magnetic behaviour. (**f**) The particles diffract as single crystals.

We speculated on the possible mechanism that results in the controlled organization of the sub-units. We performed syntheses over prolonged periods of time (up to 5 h), but did not observe particle growth (Fig. S9). Therefore, the particles form within the first hour and only the number of particles then increases with time. When magnetite was synthesized without using organic additives, the first particles (primary particles, PP) were about 2 nm in size^19^. The sizes of the sub-units in the final particles (9 ± 3 nm) differs from that of the primary particles. Therefore, the formation of our particles does not occur by simple stabilization of PPs by polyarginine, but is thought to occur in a three-stage process (Fig. 4), whereby the PPs first form and then aggregate into magnetite nanoparticles (our sub-units) with sizes of approximately 10 nm, as observed in experiments without additives^19^. This size agrees well with the sizes of the sub-units observed in the 40 nm particles. We speculate that the sub-units aggregate *via* oriented attachment. A potential reason for this behaviour is that the magnetite surface is negatively charged at the pH of our synthesis^32^ whereas the guanidine group of polyR is positively charged. As a result, polyR may serve as an “electrostatic glue” for the aggregation of the sub-units, similarly to what has been observed for ellipsoid hematite particles^33^. This electrostatic interaction, however, cannot explain the preferential aggregation along given crystallographic directions, which is the key to the single-crystal-like structure. In our case, the oriented aggregation might be favoured by the magnetic properties of the materials in the form of exchange coupling, as has also been observed for the less magnetic iron oxide hematite^34^,^35^,^36^. However, unlike synthesized hematite spindles, which had a porous structure with the magnetic properties dominated by those of the sub-units^35^, or large magnetite particles made up of misaligned particles^16^, the magnetic properties of magnetite particles synthesized in this study reflect those of the entire particle.

Neither the electrostatic interaction nor the magnetic properties can, however, explain the monodisperse character of the particles. Such monodispersity may be observed for multi-step processes, typically involving a seeding phase and a maturation phase over a prolonged period and at high temperature such as in the case of hematite^37^,^38^. These conditions, however, do not reflect those we used here. Therefore, it remains to be understood why growth is arrested here.

The uniform sizes of our particles demonstrate that the synthesis method leads to SSD magnetite particles with a narrow particle size distribution, despite the fact that the particles are made of smaller SP sub-units.

In summary, we have used 40 nm magnetite particles composed of ca. 10 nm sub-units as a model system to study the relationship between the structures and magnetic properties of nanoscale magnetite particles. We have shown that the magnetic properties of each magnetite mesocrystal are dominated by those of the superstructure. We hypothesize a three-step process for the formation of the SSD particles.

## Methods

### Synthesis of magnetite (Fe_3_O_4_) nanoparticles

Magnetite nanoparticle synthesis was performed as described previously^23^. Briefly, a computer-controlled titration device was used, consisting of a titration unit (Methrom Titrino 888) containing a 5 mL cylinder, a dosing unit (Metrohm Dosimat 805) containing a 1 ml unit and a Biotrode pH electrode. A 50 mL reaction vessel with a thermostat was used, the temperature being kept constant at 25 °C. In addition, the reactor was kept under a controlled nitrogen atmosphere. 10 mL of d.d. H_2_O was filled in the reaction vessel, to which polyarginine was added directly to reach a concentration of 0.1 mg × mL^−1^. Iron (II) chloride tetrahydrate and iron (III) chloride hexahydrate were used in a stoichiometric ratio of magnetite (Fe(II)/Fe(III) = ½) to prepare a 0.1 M iron solution. A 0.1 M NaOH solution was used for titration. Before use, all solutions were deoxygenated with nitrogen. The reaction was started by the addition of the iron solution (1 μL × ml^−1^) to the reaction vessel containing the polyarginine solution under continuous stirring using a mechanical stirrer. The pH value was kept under permanent control at pH 11 by the NaOH titration unit of the Metrohm device.

### Transmission Electron Microscopy

Low magnification bright-field (BF) images, high-resolution TEM (HRTEM) images and selected-area electron diffraction patterns were obtained at the Institute for Technical Physics and Materials Science in Budapest using Philips CM20 and JEOL 3010 TEMs at accelerating voltages of 200 and 300 kV, respectively. Images were recorded either on image plates (on the Philips CM20) or using a Gatan Orius charge-coupled device (CCD) camera (on the JEOL 3010). Digital Micrograph and SingleCrystal software was used for TEM data processing and interpretation. Particle sizes were measured from digitized images using ImageJ software.

Advanced STEM and off-axis electron holography were performed using aberration-corrected TEMs in the Ernst Ruska-Centre for Microscopy and Spectroscopy with Electrons in Forschungszentrum Jülich, Germany. A probe-aberration-corrected FEI Titan 60–300 TEM was used for obtaining BF and high-angle annular dark-field (HAADF) scanning TEM (STEM) images of the magnetite particles. The annular dark-field detector inner semi-angle was 69 mrad. Elemental maps of Fe, O and C were obtained from spectrum images acquired using both EDXS and EELS. EELS spectrum images were also used to generate thickness maps of the particles. For spectrum imaging, low-loss and core-loss energy-loss spectra were recorded simultaneously using a Gatan dual-EELS Enfinium spectrometer. The three-dimensional shapes of the particles were determined using electron tomography. HAADF STEM images were recorded at every 2° tilt in a range from +65° to −65°, using a Fischione single tilt tomography holder. The alignment of the images of and three dimensional object reconstruction was done using Tomato software^39^. To reconstruct the tomogram, a standard simultaneous iterative reconstruction algorithm (SIRT) was applied. The tomogram was visualized using an unregistered version of MeVisLab software [ www.mevislab.de].

In order to study the magnetic properties of individual magnetite particles and their chains, off-axis electron holography experiments were performed using an image-aberration-corrected FEI Titan 60–300 TEM operated at 300 kV and a Fischione dual-axis tomography holder. Electron holograms were recorded in magnetic-field-free conditions in Lorentz mode using a Gatan direct electron detection (K2-IS) camera in counting mode. This camera provides better signal-to-noise ratio and fewer pixel artefacts than a conventional CCD camera. In order to perform magnetization reversal experiments, the sample was tilted by ±75° and magnetized in the direction of the electron beam using the objective lens field of 1.41 T. The objective lens was then switched off, the sample tilted back to 0° in magnetic-field-free conditions and electron holograms of the resulting magnetic remanent states were acquired using a biprism voltage of 200 V. The sample was magnetized three times in opposite directions. Each magnetic induction map was created by adding phase contours to half of the difference between phase images recorded with the specimen magnetized in opposite directions. TEM images were processed using Gatan Digital Micrograph and Semper software. Further details of the approach used to perform off-axis electron holography experiments and analysis are given elsewhere^29^.

### Bulk Magnetic Characterization

Our earlier studies showed that during magnetite synthesis first an unknown precursor phase, possibly ferrihydrite, forms. Some of these primary precursor particles are retained in the sample even after the 40 nm magnetite particles form^19^. In order to reduce the amount of precursor particles in the material used for magnetic characterization, the samples were washed three times, with a magnet used to separate the magnetic particles from the impurities after synthesis. The supernatant was removed and the pellet was resuspended in d.d. H_2_O. In order to avoid pH changes after synthesis, d.d. H_2_O was adjusted to a pH value of 11. Two TEM samples were prepared, one from the supernatant and the other from the pellet, showing that the supernatant contained only precursor particles, whereas the pellet included the larger magnetite nanoparticles, as well as some precursor particles. Sample preparation for magnetic measurements involved drying the sample under ambient conditions and placing the dry powder in gently pressed gelatine gel caps.

The magnetite particles were characterized magnetically using hysteresis loop, backfield isothermal remanent magnetization (IRM) and first order reversal curves (FORC) using a Princeton Measurements Corporation vibrating sample magnetometer at room temperature. Hysteresis loops were measured with an average time of 300 ms. The hysteresis ratios, remanent to saturation magnetization (*Mrs*/*Ms*) and remanence coercivity to coercivity (*H*_*cr*_/*H*_*c*_) reflect the composition, particle size and chemical purity of the sample. The theoretical value for the magnetization ratio of non-interacting magnetite, dominated by magnetostatic anisotropy, is 0.5. FORCs were measured by first saturating the sample using a positive applied field of 1 T. 140 magnetization curves originating from the major hysteresis loop with a field increment of 2.3 mT and an average measurement time of 100 ms were obtained. The FORC data were transformed into a FORC diagram using codes from ref. 40 and 41. Low temperature susceptibility was measured on an AGICO MFK1-FA susceptibility bridge with the CL3 cryostat attachment, sing an applied filed of 400 A/m.

### Small Angle Neutron Scattering (SANS)

SANS measurements were performed at the instrument V4 in the Helmholtz Center Berlin, Germany. The wavelength was *λ* = 6 Å with Δλ/λ = 10%. For the experiments, circular Hellma^®^ quartz cuvettes (depth = 3.5 mm, thickness = 1 mm) filled with 300 μl of magnetite particle solution at a concentration of 46.2 mg in H_2_O were used. The 2-dimensional scattering data were corrected for detector sensitivity, and then radially integrated. Finally, the signal of an H_2_O reference sample was subtracted.

### X-ray diffraction (XRD) measurements

XRD measurements were performed using a 100 μm beam in transmission with an energy of 15 keV corresponding to the wavelength of λ = 0.82656 Å at the μ-spot beamline at the synchrotron radiation facility BESSY II (Helmholtz-Zentrum Berlin (HZB), Germany). Samples were dried on a Kapton thin film together with an α-quartz standard (NIST, standard Reference Material 1878a) as an internal quantitative XRD standard. DPDAK was used to calculate the exact sample to detector distances and the exact peak positon of the (311) peak of the sample for further lattice parameter calculations^42^,^43^. A modified Scherrer analysis was performed using a pseudo-Voigt function and including the instrumental broadening correction^43^. XRD diffractograms were compared with a magnetite reference material provided by the German Federal Institute for Materials Research and Testing (BAM).

## Additional Information

**How to cite this article**: Reichel, V. *et al*. Single crystalline superstructured stable single domain magnetite nanoparticles. *Sci. Rep.* **7**, 45484; doi: 10.1038/srep45484 (2017).

**Publisher's note:** Springer Nature remains neutral with regard to jurisdictional claims in published maps and institutional affiliations.

## Supplementary Material

Supplementary Information

Supplementary video 1

Supplementary video 2

Supplementary video 3

Supplementary video 4
