# Supplementary material for: Single crystalline superstructured stable single domain magnetite nanoparticles
Source: Sci Rep. 2017 Mar 30;7:45484. doi: 10.1038/srep45484 (PMC5371993; doi:10.1038/srep45484)
Supplement: Supplementary Information [file srep45484-s1.pdf]

Single crystalline superstructured stable single domain magnetite nanoparticles

Victoria Reichel, András Kovács, Monika Kumari, Éva Bereczk-Tompa, Emanuel Schneck, Patrick Diehle, Mihály Pósfai, Ann M. Hirt, Martial Duchamp, Rafal E. Dunin-Borkowski and Damien Faivre

Supporting Information

Supporting characterization

The magnetite particles are, as expected, composed of Fe and O, as measured by energy-dispersive X-ray spectroscopy (EDXS) (Fig. S1). In addition to the signals from the 40 nm particles, Fe and O signals are also recorded from smaller particles that are dispersed on the TEM grid (boxed area to the right of the lower part of the magnetite chain in Fig. S1d). These smaller features may represent the precursor iron oxide particles that first form during the synthesis process. The presence of C in the particles was checked in EDXS maps obtained from parts of the particles that extended above holes in the lacey C support film (without the contribution of a C-containing background, Fig. S2). The inhomogeneous distribution of the C signal observed within the particles (Fig. S2b) suggests that some C is present inside them, perhaps originating from polyarginine; however, the presence of C-bearing contamination arising from interaction between the intense and very small electron probe and the particle surface cannot be excluded. Elemental maps recorded using electron energy-loss spectroscopy (EELS) also confirmed the Fe–O composition of the particles (Fig. S3). From EELS spectrum images, it is also possible to extract information about the

relative thickness of the magnetite particles t/λ, where t is the thickness and λ the mean free path of the electrons, by taking the ratio of the integrated intensities of the zero-loss peak and the total EEL spectrum. Such a thickness map is shown in Fig. S3b. A STEM HAADF image clearly shows individual sub-units within two particles (Fig. S4a), and slices extracted from tomographic reconstructions indicate that these sub-units are not fused; instead, gaps occur between them (Fig. S4b). Electron tomographic reconstructions (Fig. S4c,d) show highly irregular surfaces, presumably resulting from the aggregation of sub-units. Selected-area electron diffraction (SAED) patterns obtained from single, aligned particles (Fig. S5) and Fourier transforms of HRTEM images (Fig. S6) reveal that the sub-units within each particle occur in a uniform crystallographic orientation. The sizes of sub-units observed in TEM images are consistent with information extracted from small angle neutron scattering (SANS) (Fig. S7) and bulk magnetic experiments (Fig. S8). According to an analysis of X-ray diffraction patterns, the mesocrystals reach their final sizes after 1 hr of synthesis (Fig. S9).

Supporting Figures

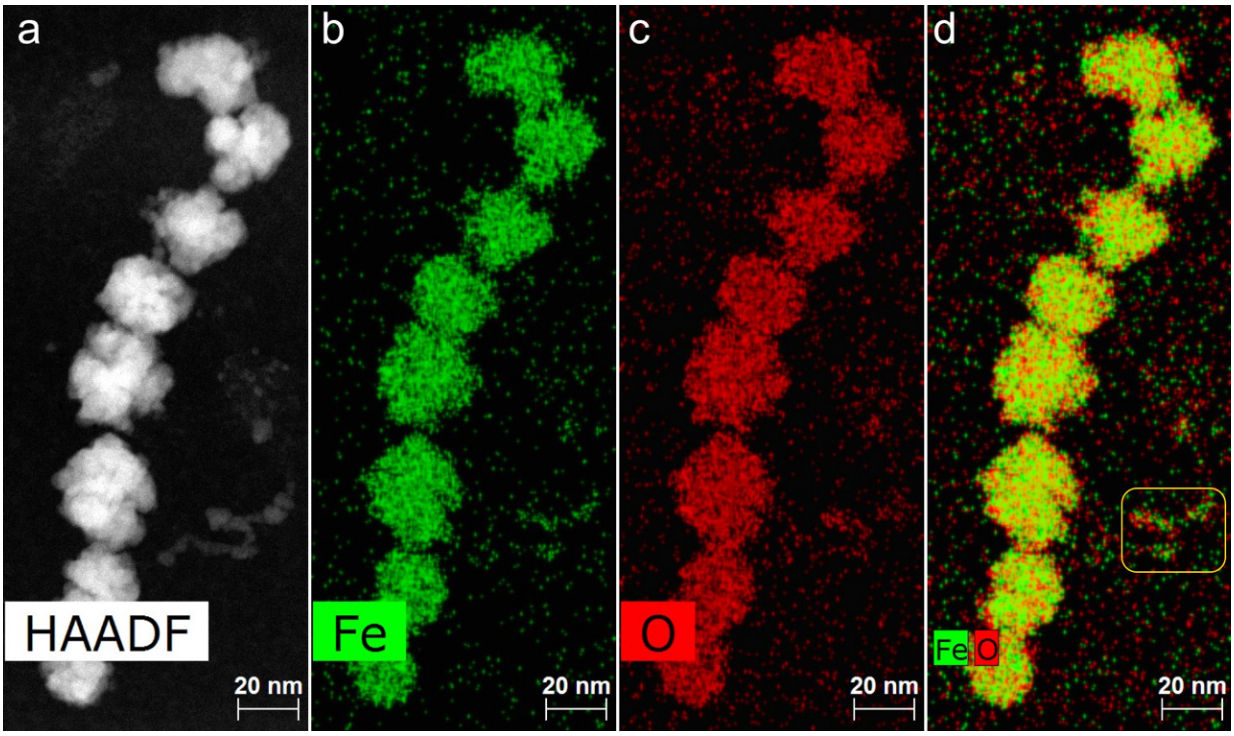

Fig. S1: Compositional analysis of a chain of eight magnetite particles. (a) HAADF STEM image; (b) Fe, (c) O, and (d) Fe+O elemental maps obtained from EDXS spectrum images. The boxed area in (d) encloses smaller particles that are also composed of Fe and O.

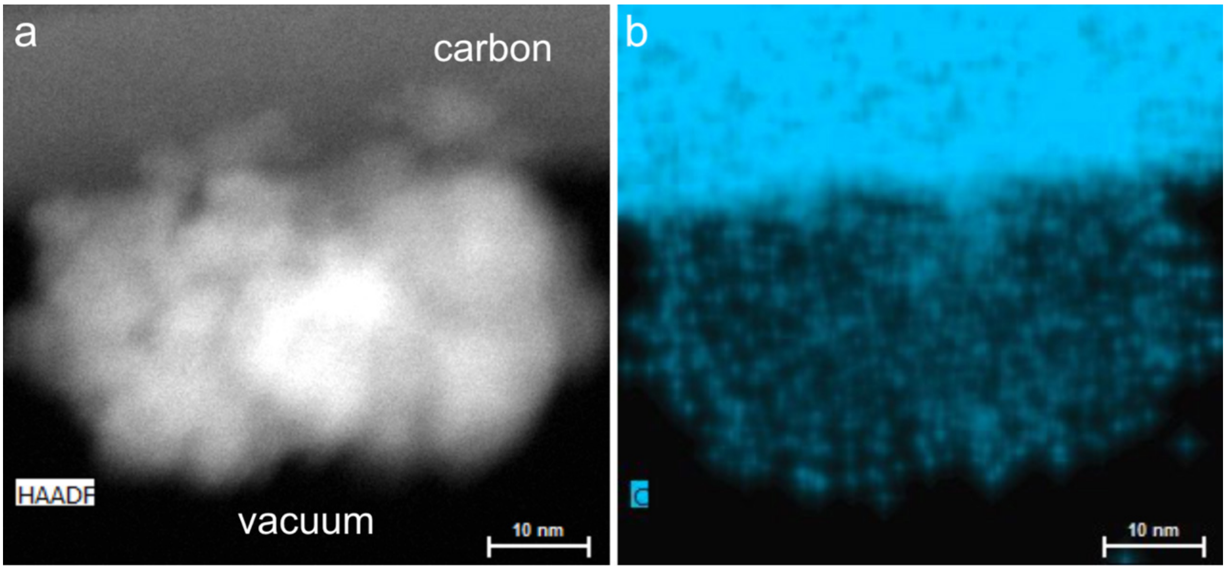

Fig. S2: Compositional analysis of a single magnetite particle attached to the side of the lacey C support film, with its lower part in vacuum. (a) HAADF STEM image and (b) C elemental map obtained from EDXS spectrum images.

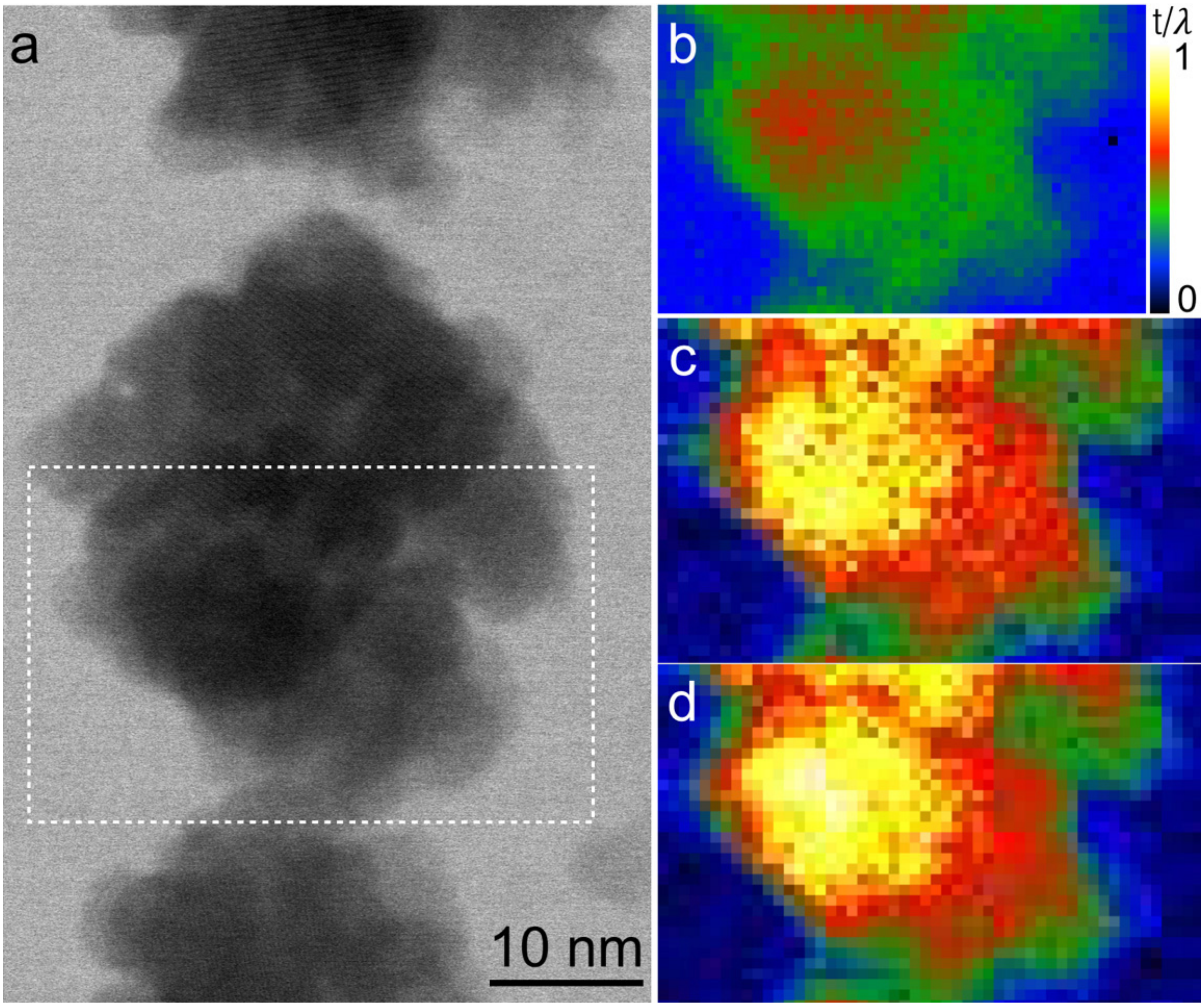

Fig. S3: Thickness and elemental distribution maps of a magnetite particle derived from a STEM EELS spectrum image. (a) BF STEM image. The area within the rectangle was used for the EELS maps. (b) Relative thickness (t/λ) of the particle. (c) Fe elemental map. (d) O elemental map.

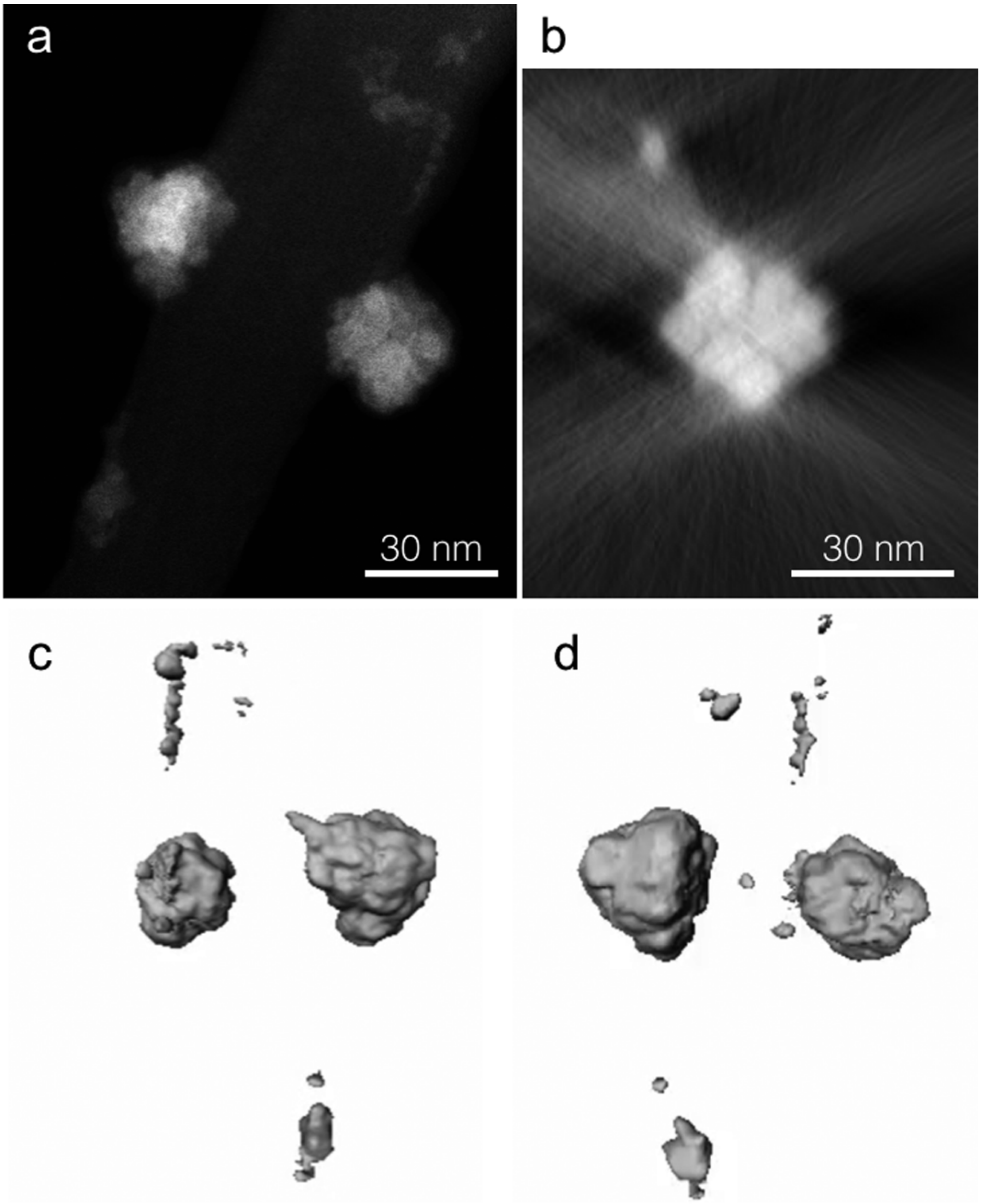

Fig. S4: (a) HAADF STEM image of two 30-nm particles and several smaller ones on carbon support. (b) Slice of the particle extracted from the tomographic reconstruction showing the ~1-nm-gap between its sub-units. (c,d) Three-dimensonal reconstruction of the particles viewed from two different angles.

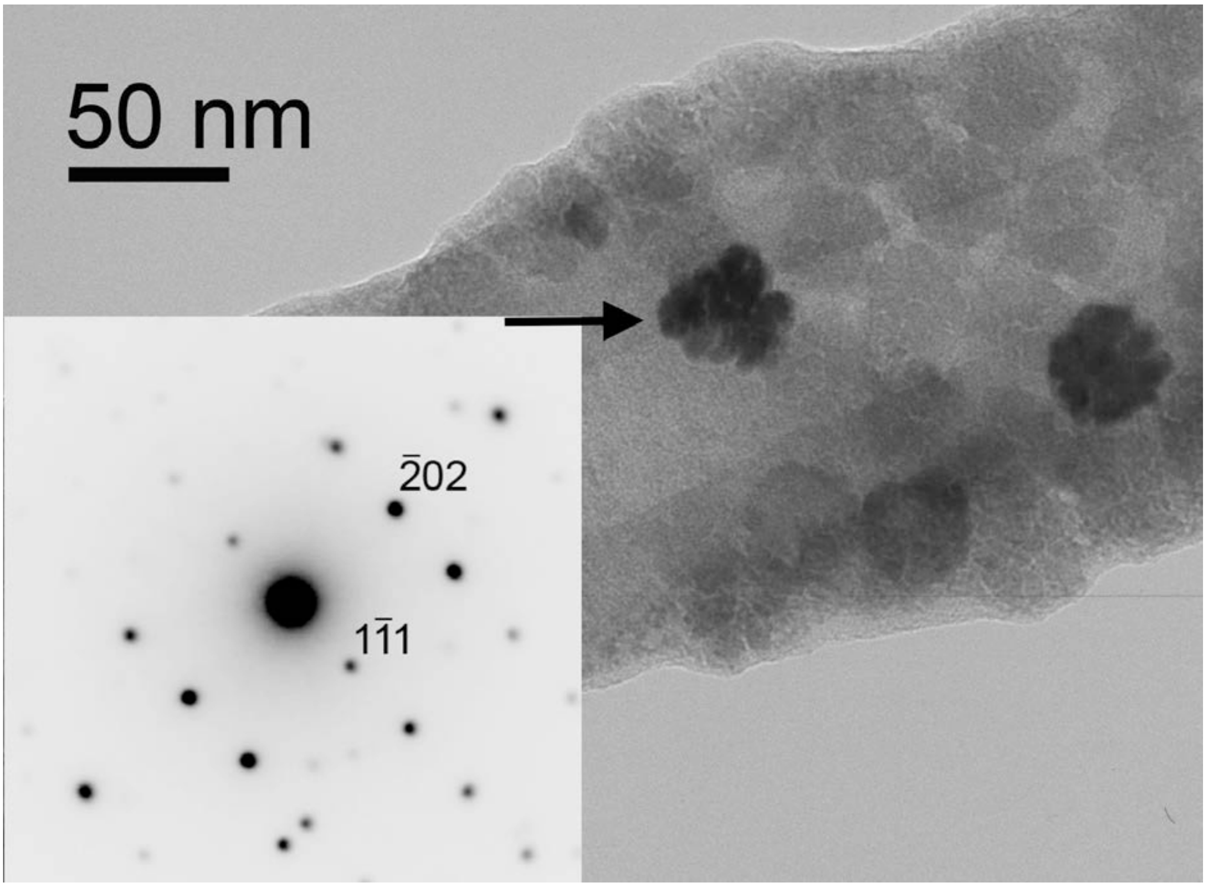

Fig. S5. Bright-field TEM image of a cluster of magnetite particles on a carbon substrate, with the inset in the lower left showing a single-crystal-like SAED pattern of the arrowed particle, obtained with the electron beam parallel to the [121] crystallographic direction of magnetite.

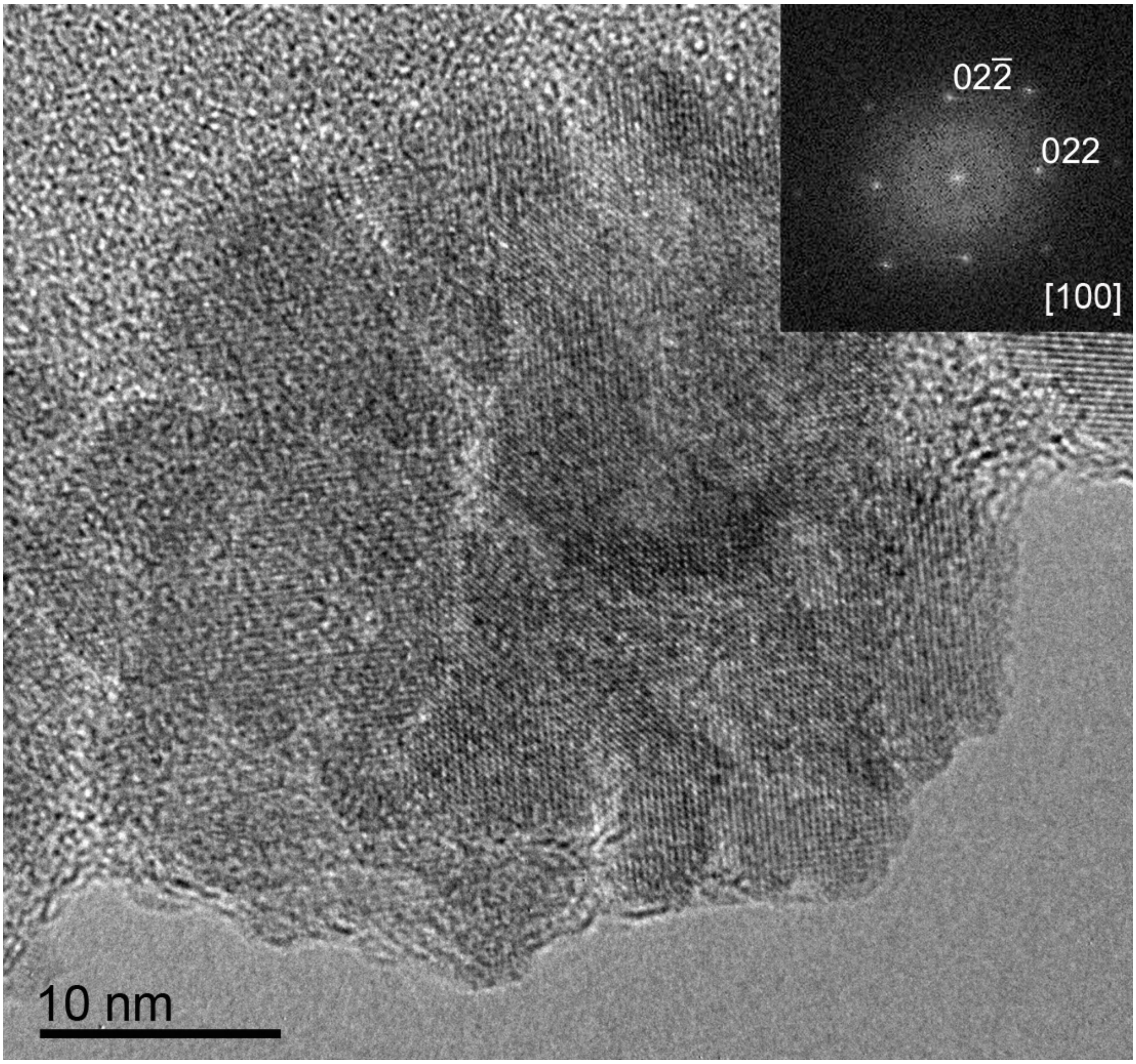

Fig. S6: HRTEM image of a magnetite particle obtained with the electron beam parallel to the [100] direction of magnetite. The Fourier transform of the particle is shown in the inset and indicates that, even though the particle consists of several small crystallites, it is in fact a single crystal. The reflections and the zone axis are indicated in the inset.

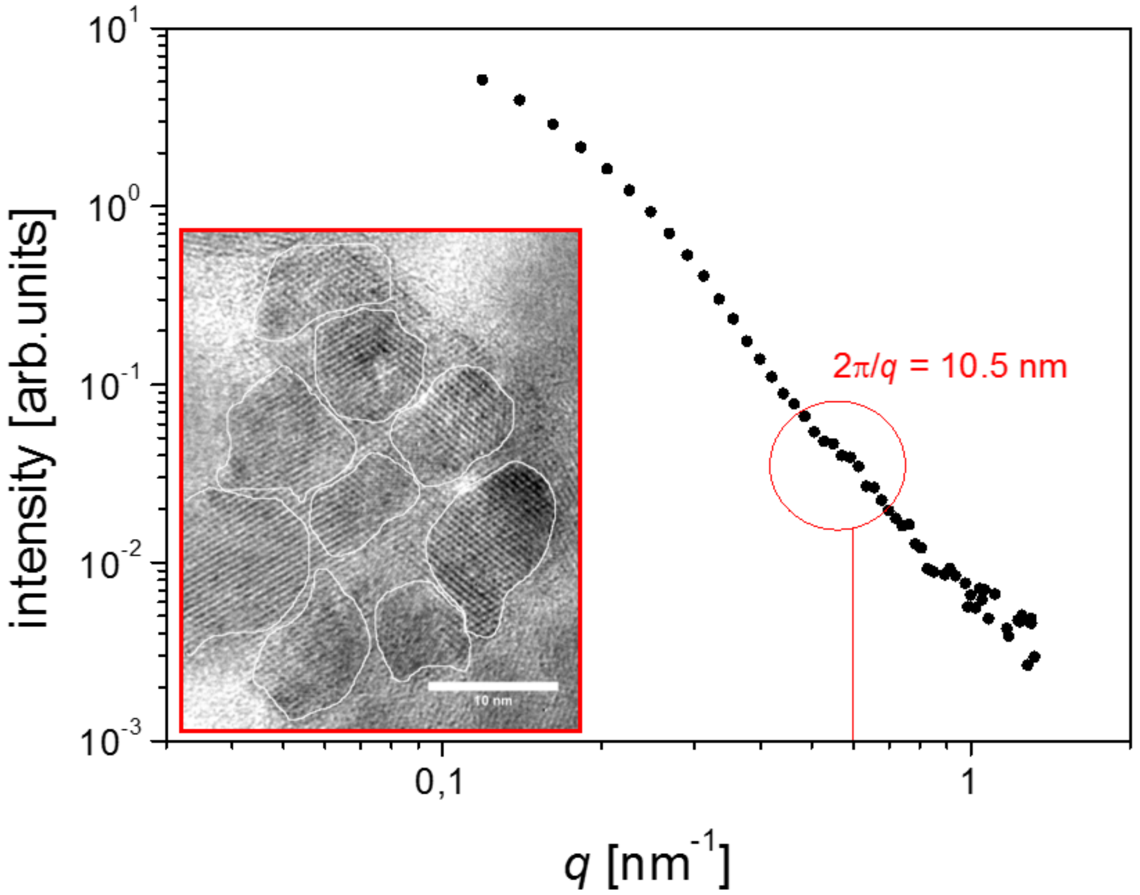

Fig. S7: Small angle neutron scattering (SANS) curve of magnetite nanoparticles exhibiting a distinct feature around a *q* value (≈ 0.6 nm^{-1}) corresponding to a characteristic length scale of 10–11 nm. This length scale is consistent with the average sub-unit size of the magnetite nanoparticles.

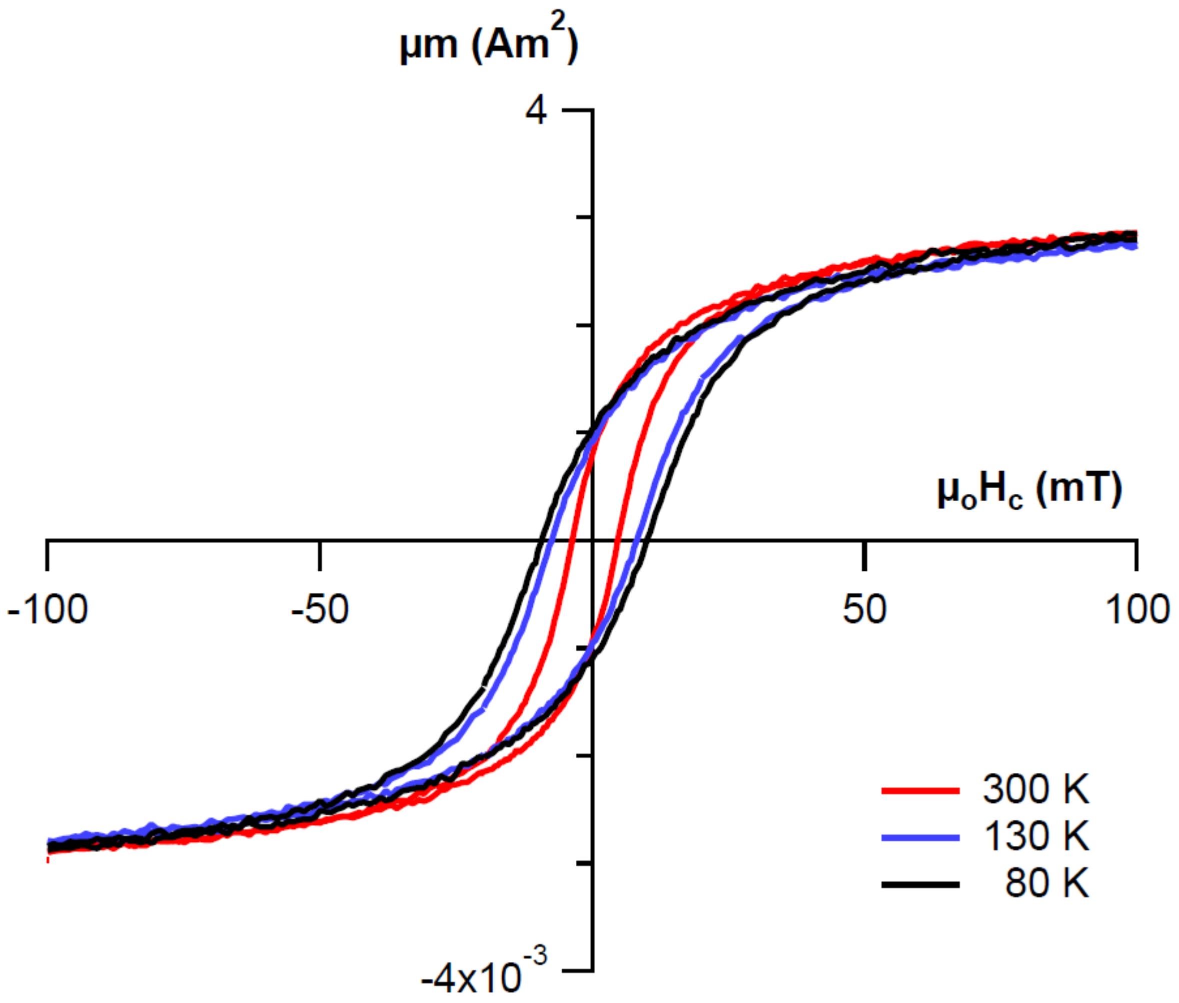

Fig. S8: Temperature-dependent magnetic characterization of the particles. The observed increase in coercivity at lower temperature is typical for SP particles.

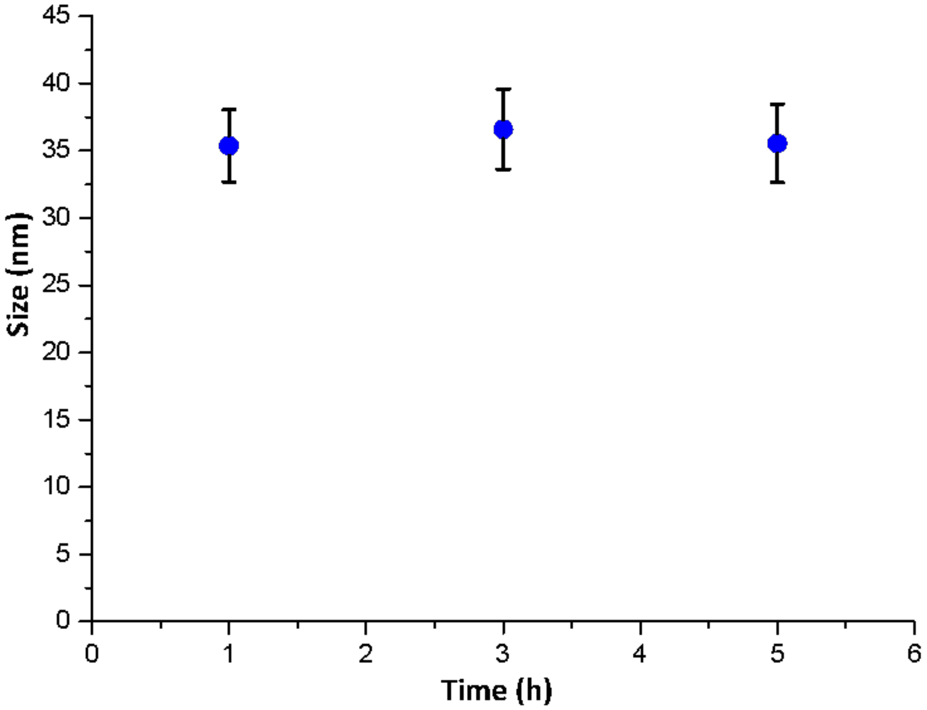

Fig. S9: Evolution of particle size during synthesis (washed samples after 1h, 3h and 5h of synthesis) as determined by using the Scherrer formula for the most intense magnetite peak in the X-ray diffractogram.

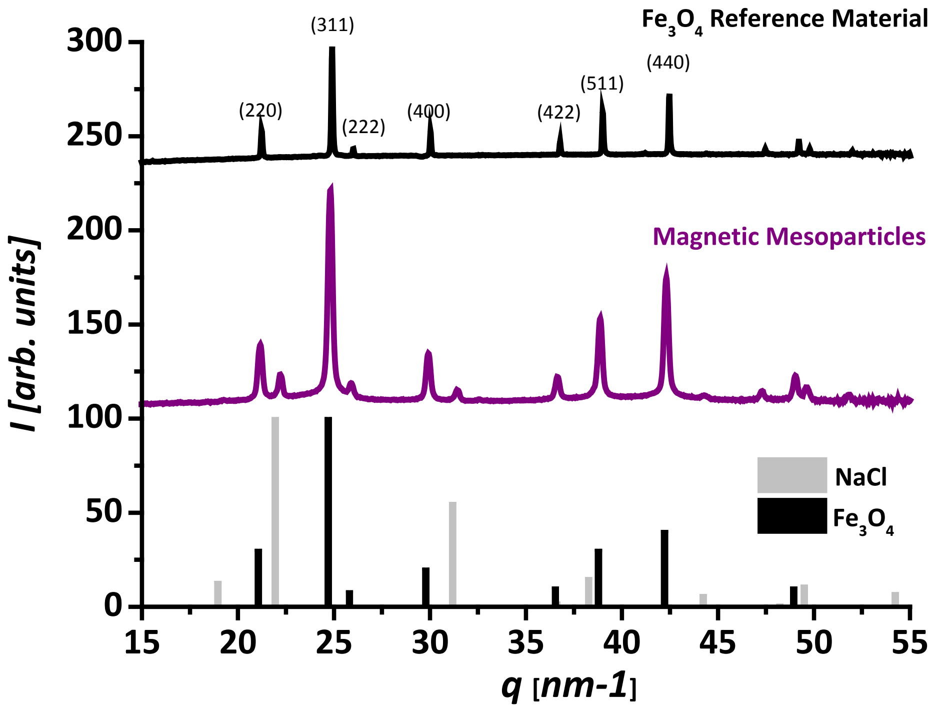

Fig. S10: X-ray diffractogram of a Fe_3O_4 reference material, the magnetic mesoparticles and theoretical diffraction peaks of Fe_3O_4 and NaCl, whereas NaCl occurs as a by-product during synthesis.

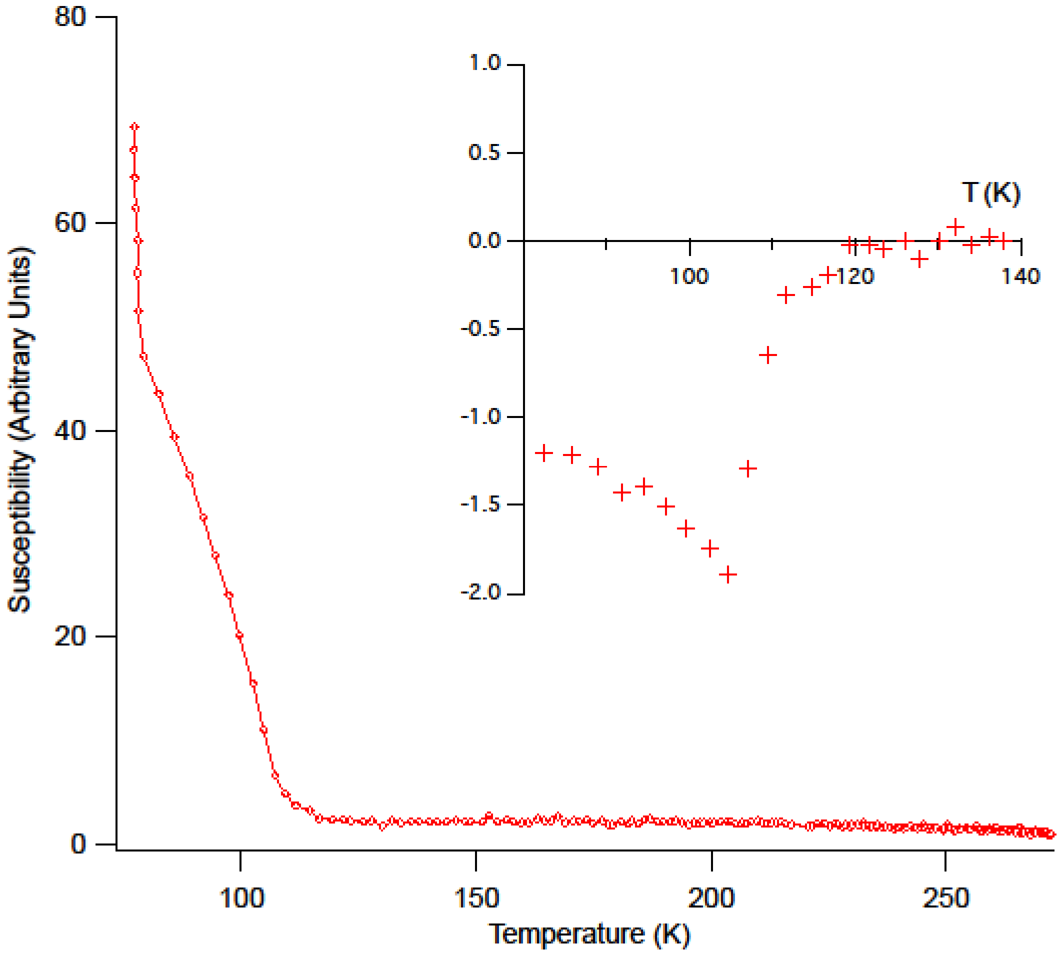

Fig. S11: Susceptibility as a function of temperature during warming form low temperature.

Description of supplementary videos

“Posfai_magnetite_mesocrystal_1”: tomographic tilt series of 3 aligned crystals

“Posfai_magnetite_mesocrystal_2”: tomographic tilt series of 2 individual crystals

“Posfai_mesocrystal1”: 3D reconstruction of data from “Posfai_magnetite_mesocrystal_1”

“Posfai_mesocrystal2”: 3D reconstruction of data from “Posfai_magnetite_mesocrystal_2”
