# Supplementary figures and images for: Single crystalline superstructured stable single domain magnetite nanoparticles
Source: Sci Rep. 2017 Mar 30;7:45484. doi: 10.1038/srep45484 (PMC5371993; doi:10.1038/srep45484)

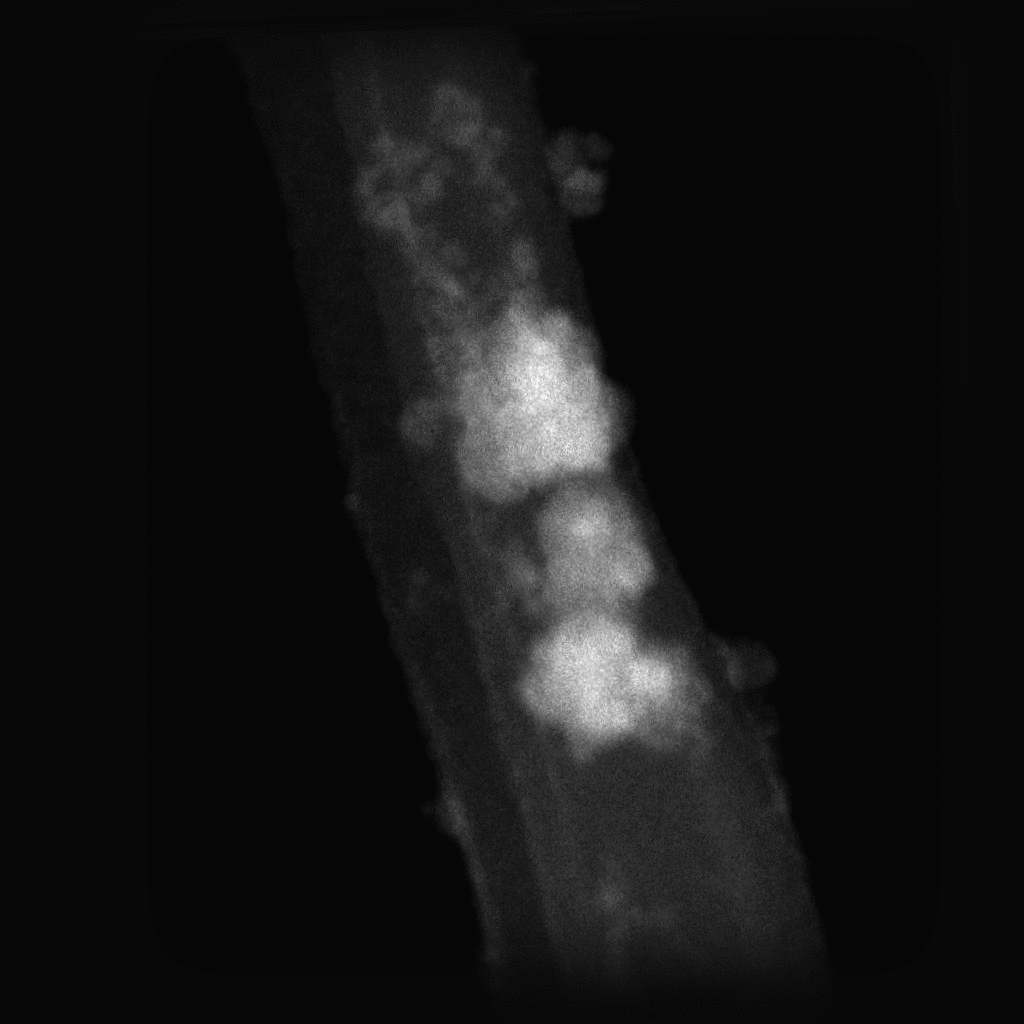

Supplement: Supplementary video 3 [file srep45484-s4.gif]

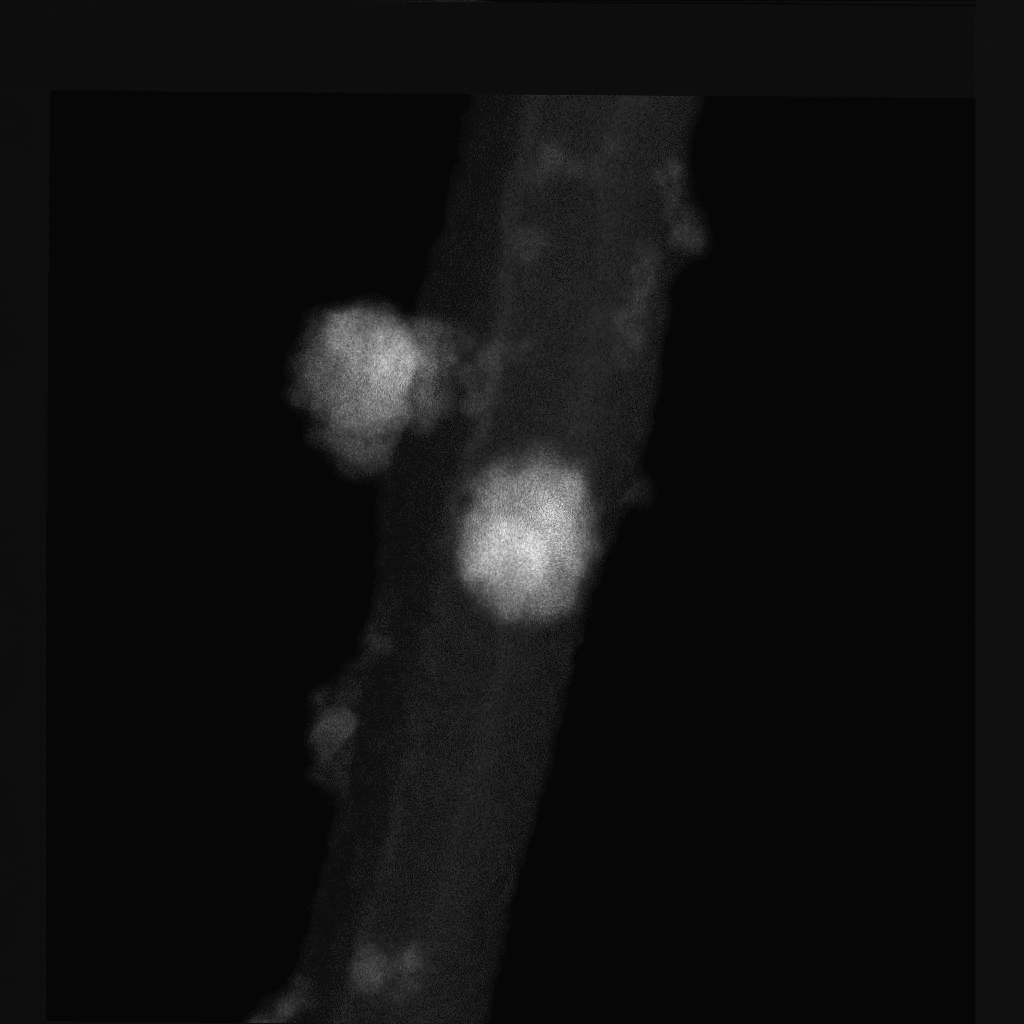

Supplement: Supplementary video 4 [file srep45484-s5.gif]
